# Draft Genome Sequence of Phytopathogenic Fungus *Fusarium fujikuroi* CF-295141, Isolated from *Pinus sylvestris*

**DOI:** 10.1128/genomeA.01164-16

**Published:** 2016-10-20

**Authors:** Michele Bertoni-Mann, Marina Sánchez-Hidalgo, Victor González-Menéndez, Olga Genilloud

**Affiliations:** Fundación MEDINA, Centro de Excelencia en Investigación de Medicamentos Innovadores en Andalucía, Parque Tecnológico Ciencias de la Salud, Granada, Spain

## Abstract

Here, we report the draft genome sequence of a new strain of *Fusarium fujikuroi*, isolated from *Pinus sylvestris*, which was also found to produce the mycotoxin beauvericin. The Illumina-based sequence analysis revealed an approximate genome size of 44.2 Mbp, containing 164 secondary metabolite biosynthetic clusters.

## GENOME ANNOUNCEMENT

The genus *Fusarium* was erected by Link in 1809 to accommodate fungi with canoe- or banana-shaped conidia (1). To date, more than 700 species have been described, constituting one of the most important phytopathogenic fungi. Around 80 *Fusarium* species are able to produce worldwide an extensive range of mycotoxins with different levels of toxicity, occurrence, and contamination.

Mycotoxins known to contaminate animal and human food include bikaverin and fusaric acid (2), fumonisins (3), beauvericin (4), and moniliformin (5). Beauvericin is a well-known cyclohexadepsipeptide mycotoxin produced by several fungal genera, such as *Beauveria, Paecilomyces, Polyporus*, and *Fusarium*. Beauvericin has insecticidal properties and can induce programmed cell death similar to apoptosis in mammalian cells, causing cytolysis accompanied by internucleosomal DNA fragmentation (6).

The strain *Fusarium fujikuroi* Nirenberg (1976) CF-295141 was isolated in our laboratory from the needles of *Pinus sylvestris* in La Hiruela (Madrid, Spain). The strain was grown in liquid MV8 culture medium for 14 days at 220 rpm, 22°C, and 70% relative humidity, and the production of beauvericin was detected in the extracts when analyzed by liquid chromatography–high-resolution mass spectrometry (LC-HRMS) analysis. *F. fujikuroi* infects a broad spectrum of crops worldwide and is responsible for high economic losses due to crop yield reduction and mycotoxin contamination. However, limited information is available regarding the diversity of *Fusarium* spp. associated with commercially propagated *Pinus* spp. or the possible diseases they cause in this tree (7).

Currently, there are five rice and maize pathogen *F. fujikuroi* genomes available in the NCBI database (8–10). This is the first genome sequencing report of a *F. fujikuroi* strain isolated from *Pinus*, which will allow whole-genome sequence comparisons between cereal- and tree-pathogenic *F. fujikuroi*, focusing on clusters of genes involved in the biosynthesis of secondary metabolites.

The *F. fujikuroi* CF-295141 genome was sequenced *de novo* using the Illumina HiSeq 2500 next-generation system at ServiceXS (Leiden, the Netherlands). Libraries were created using the NEBNext Ultra DNA library prep kit (New England Biolabs). The quality and yield after sample preparation were measured with the fragment analyzer (AATI), and the size of the resulting product was consistent with the expected size of 500 to 700 bp. A concentration of 15.0 pM DNA was used. A short-read genome assembler based on de Bruijn graphs with k-mer size of 64 was used for assembly. Contigs shorter than 200 bp were removed from the assembly. The assembled genome resulted in 237 contigs. The *N*_50_ was 850,952 bp, and the maximum contig length was 2,003,258 bp. The total genome size was 44,205,264 bp (G+C content, 48.2%) at 100× coverage.

The assembled genome was used to assess the biosynthetic potential of this fungus using the Antibiotics and Secondary Metabolite Analysis Shell (antiSMASH) (11). The genome contained 164 secondary metabolite synthesis clusters, including those coding for 12 polyketides, 14 nonribosomal peptides, 11 terpenes, and six hybrid compounds.

### Accession number(s).

This whole-genome shotgun project has been deposited at DDBJ/ENA/GenBank under the accession no. MBPS00000000. The version described in this paper is version MBPS01000000.
