# Correction for Bertoni-Mann et al., “Draft Genome Sequence of Phytopathogenic Fungus *Fusarium proliferatum* CF-295141, Isolated from *Pinus sylvestris*”

**DOI:** 10.1128/genomeA.01116-17

**Published:** 2017-10-26

**Authors:** Michele Bertoni-Mann, Marina Sánchez-Hidalgo, Victor González-Menéndez, Olga Genilloud

**Affiliations:** Fundación MEDINA, Centro de Excelencia en Investigación de Medicamentos Innovadores en Andalucía, Parque Tecnológico Ciencias de La Salud, Granada, Spain

## AUTHOR CORRECTION

Volume 4, no. 5, e01164-16, 2017, https://doi.org/10.1128/genomeA.01164-16. Page 1: The article title should read as given above, and the fungal strain should be identified throughout as *Fusarium proliferatum* CF-295141 rather than *Fusarium fujikuroi* CF-295141.

